# Shared Neural Computations for Syntactic and Morphological Structures: Evidence From Mandarin Chinese

**DOI:** 10.1111/cogs.70220

**Published:** 2026-05-12

**Authors:** Xinchi Yu, Sebastián Mancha, Xing Tian, Ellen Lau

**Affiliations:** ^1^ Program in Neuroscience and Cognitive Science University of Maryland; ^2^ Department of Linguistics University of Maryland; ^3^ Division of Arts and Sciences New York University Shanghai; ^4^ Shanghai Key Laboratory of Brain Functional Genomics (Ministry of Education), School of Psychology and Cognitive Science East China Normal University; ^5^ NYU‐ECNU Institute of Brain and Cognitive Science at NYU Shanghai

**Keywords:** Syntactic structure, Morphological structure, Non‐lexicalism, Lexicalism, Language comprehension

## Abstract

Although psycho‐/neuro‐linguistics has assumed a distinction between morphological and syntactic structure building as in traditional theoretical linguistics, this distinction has been increasingly challenged by theoretical linguists in recent years. Opposing a sharp, lexicalist distinction between morphology and syntax, non‐lexicalist theories propose common morpho‐syntactic structure‐building operations that cut across the realms of “morphology” and “syntax,” which are considered distinct territories in lexicalist theories. Taking advantage of two pairs of contrasts in Mandarin Chinese with desirable linguistic properties, namely, compound versus simplex nouns (the “morphology” contrast, differing in morphological structure complexity per lexicalist theories) and separable versus inseparable verbs (the “syntax” contrast, differing in syntactic structure complexity per lexicalist theories), we report one of the first pieces of evidence for shared neural responses for morphological and syntactic structure complexity in language comprehension, supporting a non‐lexicalist view where shared neural computations are employed across morpho‐syntactic structure building. Specifically, we observed that the two contrasts both modulated neural responses in left anterior and centro‐parietal electrodes in an a priori 275:400 ms time window, corroborated by topographical similarity analyses. These results serve as preliminary yet *prima facie* evidence toward shared neural computations across morphological and syntactic structure building in language comprehension.

## 1 Introduction

Both in speech and in text, language comprehension requires inferring and building up the linguistic structures intended by the speaker or writer on the basis of the sensory evidence they provide. According to the traditional lexicalist view, the linguistic structures include, for example, morphological structures (structures within words with “morpheme” as their unit) and syntactic structures (structures over phrases in a sentence with “word” as their units). Is there really a sharp distinction between “sentence structure” versus “word structure” (according to lexicalist theories) in language comprehension, or is syntax “all‐the‐way‐down,” removing the need to posit a separate set of word‐forming “morphological” operations (according to non‐lexicalist theories; Krauska & Lau, 2023)? Although the psycho‐/neuro‐linguistics literature traditionally studies morphology and syntax separately following the earlier morphology–syntax distinction in theoretical linguistics (Chomsky, 1970; Lapointe, 1980; Williams, 1981), a growing body of literature in theoretical linguistics challenges the idea that so‐called “morphological” and “syntactic” rules/operations are qualitatively distinct (Embick, 2015; Halle & Marantz, 1993; Harley, 2014; Noyer, 1998; Preminger, 2021; Siddiqi, 2010). This theoretical trend manifests in both generative and construction grammar traditions: for example, lines of work closely related to construction grammar also challenges a strict lexicon‐grammar distinction (Booij, 2010; Culicover & Jackendoff, 2005; Jackendoff, 2007; Piñango, 2022). A non‐lexicalist architecture is also echoed in the tree‐adjoining grammar tradition (Joshi & Schabes, 1997), where, for example, morphological primitives such as verbal inflections exist alongside phrasal syntax at the elementary tree level (Frank, 2004, 2010). However, there has been little psycho‐/neuro‐linguistic evidence on this issue (see Krauska, 2024; Krauska & Lau, 2023).

**Fig. 1:**
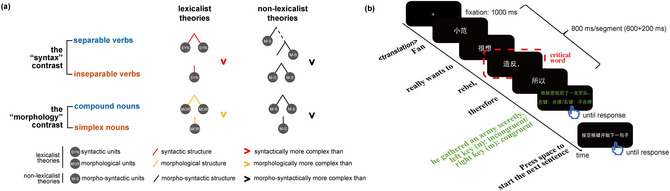
(a) Illustration of the two pairs of conditions (i.e., two contrasts) that we employed in the current study. According to lexicalist theories, separable verbs are more complex than inseparable verbs in syntactic structure, while compound verbs are more complex than simplex nouns in morphological structure. Meanwhile, according to non‐lexicalist theories, these two complexities are not qualitatively different (i.e., they are both morpho‐syntactic in nature). Note that the exact shape of the trees may differ according to different theoretical linguistic theories, yet the complexity difference in the two contrasts holds true for all major relevant theories that we are aware of. (b) An illustration of a trial in our current study. English translation is provided below the time arrow.

A natural thought then would be to contrast conditions that only differ in morphological structure complexity, and conditions that only differ in syntactic structure complexity, and examine if they elicit similar neural responses. However, there are inevitable and widely known challenges in manipulating linguistic structure without simultaneously altering conceptual structure and/or phonological structure (Călinescu, Ramchand, & Baggio, 2023; Pylkkänen, 2019). For example, *red boat* is indeed syntactically more complex than *xkq boat* (where *xkq* is a list of meaningless consonant letters), but the former has a more complex semantic‐conceptual structure as well (Bemis & Pylkkänen, 2011, 2013). Among recent work in other languages proposing encouraging solutions to this problem with interesting linguistic properties in those languages (Flick & Pylkkänen, 2020; Law & Pylkkänen, 2021; Matar, Dirani, Marantz, & Pylkkänen, 2021), in the current paper, we take advantage of the desired properties of “separable verbs” in Mandarin Chinese—compound forms whose meanings are non‐compositional (e.g., *zao4fan3* means “rebel,” where *zao4* means “manufacture” and *fan3* means “reverse”) but which, unlike compounds in English, can be syntactically separated while preserving their non‐compositional meaning. This class of compounds contrasts with another set of “inseparable” verb compounds, which are matched in conceptual complexity as well as form complexity but which cannot be separated. At the same time, we also included a second set of conditions in which we compared the response to (inseparable) compound nouns with simplex (i.e., “monomorphemic”) nouns. According to lexicalist theories, separable verbs and inseparable verbs (the syntax contrast) only differ systematically in syntactic structure complexity, while compound nouns and simplex nouns (the morphology contrast) only differ systematically in morphological structure complexity; while non‐lexicalist theories would posit that the two types of complexities are qualitatively the same. By comparing the responses across the two pairs of conditions (Fig. 1a; Table 1), we can ask whether the neural signature for a “syntactic” contrast is qualitatively similar to a “morphological” contrast in the same participants. Of course, given the limited spatial resolution of electroencephalography (EEG), we cannot guarantee that the neural bases are exactly the same for even the same scalp distributions (which is the case for nearly all neuroimaging methods apart from perhaps single‐neuron recording). Here, we are only trying to determine if there is *prima facie* evidence for shared neural responses or if it is the case that even such preliminary evidence is hard to obtain. Our current study should be considered a stepping‐stone toward eventually answering this research question instead of the final say.

**Table 1:** Examples of the separable verbs, inseparable verbs, compound nouns, and simplex nouns that we used in the current experiment

Contrasts	Experimental Conditions	Examples	Pinyin Transcriptions	Meaning	Properties of Note
**The syntax contrast**: **separable verb** **minus** **inseparable verb**	**Separable verb**	造反	zao4fan3	[manufacture]‐[reverse], “rebel”	Syntactically separable Non‐compositional in meaning, syntactically intransitive, disyllabic
		跑步	pao3bu4	[run]‐[step], “jog”	
		退休	tui4xiu1	[back]‐[rest], “retire”	
	**Inseparable verb**	迟疑	chi2yi2	[late]‐[doubt], “hesitate”	Syntactically inseparable Non‐compositional in meaning, syntactically intransitive, disyllabic
		社交	she4jiao1	[society]‐[communicate], “socialize”	
		败北	bai4bei3	[fail]‐[north], “lose (in wars)”	
**The morphology contrast**: **compound noun** **minus** **simplex noun**	**Compound noun**	热狗	re4gou3	[hot]‐[dog], “hotdog”	2 morphemes Non‐compositional in meaning, disyllabic
		大衣	da4yi1	[big]‐[clothes], “overcoat”	
		酸奶	suan1nai3	[sour]‐[milk], “yoghurt”	
	**Simplex noun**	披萨	pi1sa4	“pizza”	1 morpheme, transliteration‐based loan words Non‐compositional in meaning, disyllabic
		夹克	jia2ke4	“jacket”	
		拿铁	na2tie3	“latte”	

### 1.1 Separable and inseparable verb compounds in Mandarin Chinese

The current study employs an intriguing class of verb compounds in Mandarin Chinese, which can be syntactically separated while preserving their non‐compositional compound meaning. Here, we will use the term “separable verbs,” but in the literature, they go by many other names as well, including “ionized forms” (Chao, 1968), “separable words” (Han & Wang, 2022), and “splittable compounds” (Lu, Dai, & Wu, 2022; Siewierska, Xu, & Xiao, 2010), as well as its Chinese name *liheci* (离合词, Petrovčič, 2016; Ye & Pan, 2023).

The separable verb phenomenon has been well‐documented and extensively discussed in the theoretical linguistics literature (Chan, 2016; Chao, 1968; Huang, 1984; Li & Thompson, 1981; Packard, 2000; Siewierska et al., 2010; Wang, 2011; Xue, 2002; Ye & Pan, 2023; Yeh, 2020; Zhang, 2007; Zhu & Liu, 2020), and somewhat similar phenomena have also been documented in other languages, such as Vietnamese (Nhàn, 1984; Noyer, 1998) and Cantonese (Chan & Cheung, 2021; Yip, Lee, & Chan, 2021). An example is illustrated in (1). For the separable verb of “造反” (zao4fan3, [manufacture]‐[reverse], “rebel”), syntactic units (e.g., aspect markers, classifiers, quantifiers, resultative verb complements, Siewierska et al., 2010; Wang, 2011; Ye & Pan, 2023) can be inserted between the two morphemes, but the meaning remains non‐compositional (the compound meaning is not transparently derivable as a function of its parts, and thus the compound meaning must be acquired by learners separately from the meaning of its parts). By analogy, if “babysit” in English were a separable compound, one could syntactically separate its parts—for example, “baby‐ one time ‐sit”—while preserving the non‐compositional meaning^1^,

(1) 造   了    很    多   次   反
 zao4   le0    hen3  duo1   ci4  fan3
 
zao4  ASP  very   many  CL  fan3
 “rebelled many times”

One of the primary reasons that separable verbs have engendered so much interest in the literature is because they seem to put pressure on a traditional notion of “word” as a stored object of semantic, syntactic, and phonological form that enters into semantic, syntactic, and phonological relations with other words. Standard, inseparable compounds like “seahorse” may seem to suggest a non‐isomorphic pairing of a single concept to multiple form parts, but these cases can still be accommodated in a traditional framework by assuming that “seahorse” is a single phonological form, which corresponds to a single element in the syntactic structure and which in turn maps to a single concept. Separable verbs cannot be handled this way because they manifestly demonstrate multiple elements that can be manipulated by the syntax but which realize the single stored (non‐compositional) meaning.

The dissociation between conceptual complexity and syntactic complexity evident in Chinese separable verb compounds provides a novel opportunity for investigating the neural correlates of syntactic structure complexity in comprehension as dissociated from the complexity of conceptual structures. In the current study, we do this by comparing the neural response to separable verb compounds with that of a different syntactic class of verbal compounds, which, like English compounds, are “inseparable.” For example, in the case of the inseparable verb of “迟疑” (chi2yi2, [late]‐[doubt], “hesitate”), no syntactic units can be inserted in between the two parts as illustrated in (2a). Instead, one has to use an expression like the one indicated in (2b). For more examples of separable and inseparable verbs we used, see Table 1 (for a full list, see our open data).

(2a) *迟   了   很   多   次   疑
 chi2   le0    hen3  duo1  ci4  yi2
 
chi2  ASP  very  many  CL  yi2
 Intended: “hesitated many times”

(2b) 迟   疑   了     很   多   次
 chi2   yi2   le0   hen3  duo1  ci4
 
chi2 yi2  ASP  very  many  CL
 “hesitated many times”

Both separable and inseparable verbs used in the current experiment consist of two morphemes, are non‐compositional in meaning, and are syntactically intransitive (i.e., cannot take direct objects syntactically). Therefore, we can assume that the primary systematic difference between the separable versus inseparable verbs we used here is in their syntactic representation.^2^ We note that different syntactic theories vary in exactly how that syntactic difference is realized. In standard lexicalist theories, inseparable compound verbs are considered to consist of a single syntactic unit (i.e., one “word”; Chung, 2006), and separable compound verbs are taken rather to consist of a complex syntactic structure relating multiple syntactic units (Badan, 2013; Chan, 2016; Guo, 2017; Huang, 1997; Pan & Ye, 2015). Non‐lexicalist theories, on the other hand, allow the possibility that inseparable compound verbs also have internal syntactic parts corresponding to their compound phonological form parts. Such theories could posit instead that the ability or inability to be syntactically separated is driven by the two compound classes having distinct kinds of syntactic structure (cf., Zhang, 2007). Regardless of which analysis turns out to be correct, what is important for our first comparison is just that the two types of compounds differ underlyingly in their syntactic representation even when they are not actually separated, and thus that differential neural responses to these two compound types can be attributed to syntactic inference processes.

### 1.2 Compound and simplex nouns in Mandarin Chinese

Although Chinese is sometimes mistakenly characterized as a language “without much morphology,” in fact compound forms dominate the vocabulary of modern Chinese, in some estimates as much as 88% of Chinese lexicon (Zhou & Marslen‐Wilson, 1994). Apart from separable versus inseparable verbs that differ in syntactic structure complexity, in this study, we also introduced a second pair of conditions in which the items differ in morphological structure complexity but do not differ in their ability to be visibly separated in the syntax. This second comparison made use of transliteration‐based loanwords in Mandarin like *pi1sa4* (from “pizza”), which are unambiguously simplex (monomorphemic according to lexicalist theories; cf., Hsu, Pylkkänen, & Lee, 2019; Wei, Niu, Taft, and Carreiras, 2023). We compared the event‐related potential (ERP) response to these simplex nouns with inseparable compound nouns to yield what would be traditionally considered a “morphological structure” contrast, and we then conducted a topographical similarity analysis to evaluate whether this effect is qualitatively similar to the “syntactic structure” contrast between separable and inseparable verbs, as might be predicted by “syntax‐all‐the‐way‐down” theories. Note that by the catchphrase of “syntax all the way down,” we refer generally to theories that propose shared operations and mechanisms across morphology and syntax, and we do not intend to imply, for example, that syntax necessarily “dominates” morphology.

In the current study, we focused on ERP responses in the 275:400 ms time‐window associated in prior literature with LAN (left anterior negativity) modulations of syntax and morphology. Particularly relevant for our current study, Y. Wei et al. (2023) reported results from a contrast between Mandarin compounds and simplex words (with a variety of part‐of‐speech, including loanword nouns as in our current study) showing an increased LAN response for compounds relative to simplex words in the 275:400 ms time window. Although we similarly expected to see differential effects of structure over left anterior electrodes in the current study, we chose to be cautious here by testing for effects in multiple regions across the head because the EEG system used in this study required a different referencing technique (an average reference) that could potentially cause differences in scalp distribution. Complementary to this analysis, we also employed topographical similarity analyses to test whether and when the two complexities modulate scalp distribution in similar ways. Since prior studies suggested that the concreteness of nouns could also modulate anterior channels in a similar time window (where more concrete nouns are more negative than less concrete nouns in amplitude; Adorni & Proverbio, 2012; Barber, Otten, Kousta, & Vigliocco, 2013; Zhang, Guo, Ding, & Wang, 2006), participants also rated the concreteness of compound and simplex nouns after the experiment. In the current study, we embedded all linguistic expressions in relatively neutral sentential contexts (Table 2), in order to ensure more realistic processing compared to isolated‐word paradigms (see Section 3.2).

**Table 2:** Example sentential stimuli for our experiment; the critical segment (i.e., the “critical word”) for our analysis is marked in bold

**Compound and Simplex Nouns**
**Structure of Sentences**
[Because] **[compound/simplex noun]** [is] [segment 4] [segment 5,] [therefore/so] [continuation.] [因为] **[compound/simplex noun]** [是] [segment 4] [segment 5,] [所以/因此] [continuation.]
**Example**:
因为/**热狗**/是/一种/高热量的/食物, /所以/不是很健康。 Because/**hotdog**/is/a.kind.of/high‐calorie/food,/therefore/not.very.healthy. “Because hotdog is a kind of high‐calorie food, therefore it is not very healthy.”
**Separable and inseparable verbs**
**Structure of sentences**
[Name] [wants to/does not want to/really wants to] **[separable/inseparable verb,]** [so/therefore] [continuation.] [Name] [想要/不想/很想] **[separable/inseparable verb,]** [所以/因此] [continuation.]
**Example**:
小范/很想/**造反**, /所以/他秘密组织了一支军队。 Fan[name]/really.wants.to/**rebel,**/therefore/he.gathered.an.army.secretly. “Fan really wants to rebel, therefore he gathered an army secretly.”

## 2 Results

### 2.1 Behavioral results

Accuracy for the main experiment: The analyzed subjects generally had a high accuracy in the continuation congruity judgements (mean 96%, range 85%–100%), suggesting that they were attentively comprehending the sentences.

Post‐test ratings: The separability ratings results confirmed that the separable verbs we used were indeed more separable than the inseparable verbs (separable: 6.1; inseparable: 2.5; *t*(23) = 20.15, *p* < .001, two‐tailed). For the concreteness ratings, all item types received relatively high concreteness ratings, but we did observe a numerically small but significant difference between conditions (compound: 6.3, simplex: 6.2, *t*(23) = 3.93, *p* < .001, two‐tailed). For details of these post‐tests, see the Methods section.

### 2.2 ERP analysis: The LAN time window (275:400 ms)

**Fig. 2:**
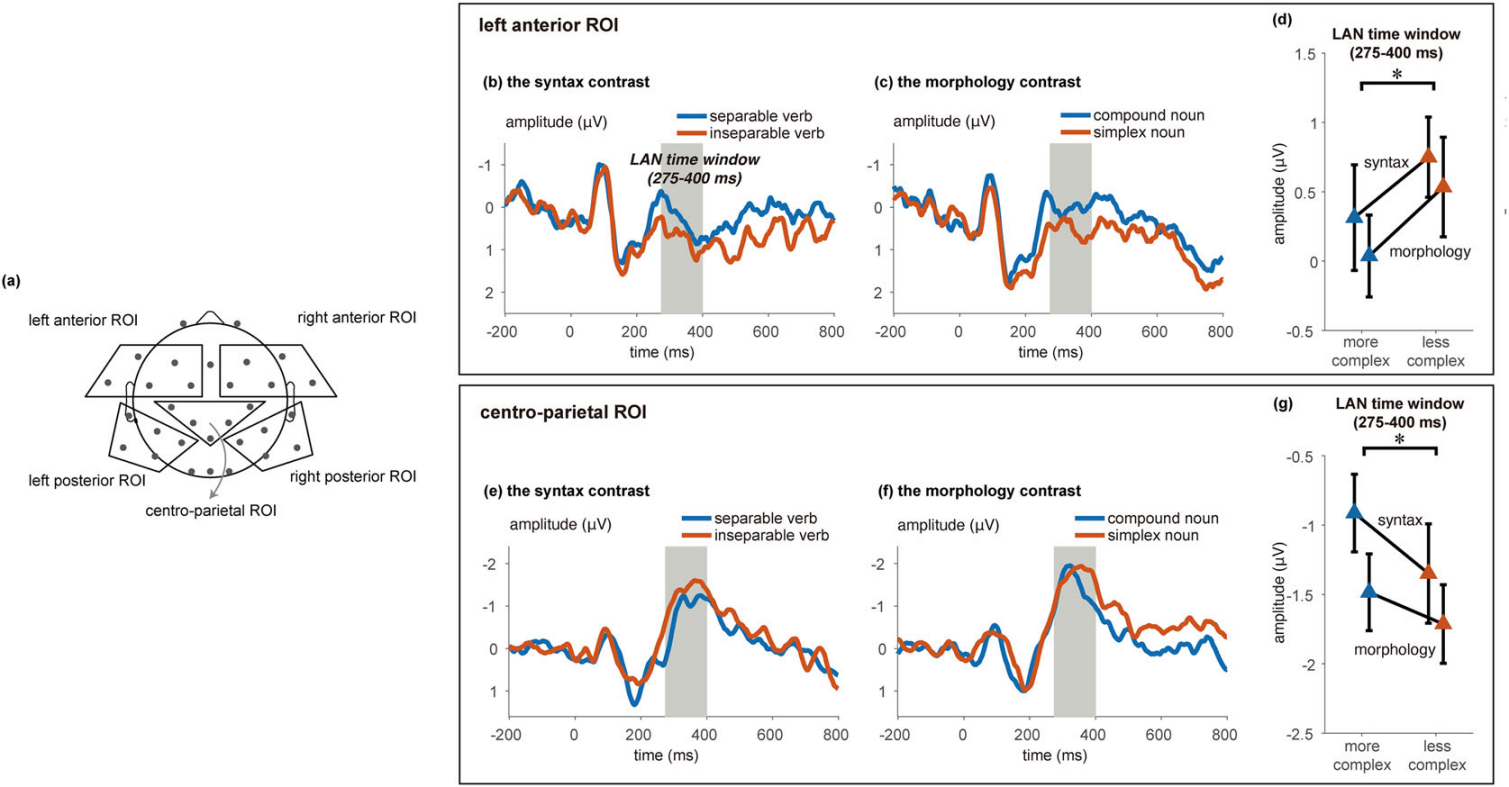
Event‐related potential (ERP) results. Region of interest (ROI) definitions are illustrated in (a), and the right panels illustrate the ERPs for all four conditions in the two ROIs, which showed significant effects of complexity. (a) The ROIs (regions of interest) in our current study. The left anterior ROI consists of F3, F7, FC5, FC7, and FT9; the right anterior ROI consists of F4, F8, FC2, FC6, and FT6; the centro‐parietal ROI consists of CP1, CP2, Pz, C3, and C4; the left posterior ROI consists of T7, CP5, P7, P3, and TP9; the right posterior ROI consists of T8, CP6, T8, P4, and TP9. (b) Experimental conditions. (c) Grand average ERPs within the left anterior ROI −200:800 ms around the onset of critical word for separable verbs and inseparable verbs. The a priori time window of 275:400 ms is highlighted in gray. (d) Grand average ERPs within the left anterior ROI −200:800 ms around the onset of critical word for compound nouns and simplex nouns. The a priori time window of 275:400 ms is highlighted in gray. (e) Mean response and standard error within the 275:400 time window in the left anterior ROI for the “syntax” and “morphology” contrasts (*: *p* < .05). (f) Grand average ERPs within the centro‐parietal ROI for compound nouns and simplex nouns. (g) Grand average ERPs within the centro‐parietal ROI for separable verbs and inseparable verbs. (h) Mean response and standard error within the 275:400 time window in the centro‐parietal ROI for the “syntax” and “morphology” contrasts (*: *p* < .05). For the ERP waveforms of all scalp electrodes, see Supporting Information.

We conducted an omnibus repeated‐measure ANOVA analysis, with region of interest (ROI; five ROIs, Fig. 2a), contrast type (the morphology contrast, the syntax contrast), and complexity (more complex, less complex morpho/syntactic structure) as independent variables, and the mean response within the 275:400 ms time window in each ROI as the dependent variable (thus, a 5 × 2 × 2 three‐way ANOVA). Namely, the compound noun condition corresponds to the contrast type of “morphology” and a complexity of “more complex,” the simplex noun condition corresponds to “morphology” and “less complex,” the separable verb condition corresponds to “syntax” and “more complex”, and the inseparable verb condition corresponds to “syntax” and “less complex” (Fig. 2b).

The three‐way ANOVA revealed a significant main effect of ROI, *F*(2.07,47.55) = 8.50, *p* < .001, a marginally significant interaction effect between contrast type and ROI, *F*(2.76,63.58) = 2.46, *p* = .076, and critically a significant interaction effect between complexity and ROI, *F*(2.90,66.59) = 2.77, *p* = .050; all other effects were not statistically significant (*p*s > .21).

**Fig. 3:**
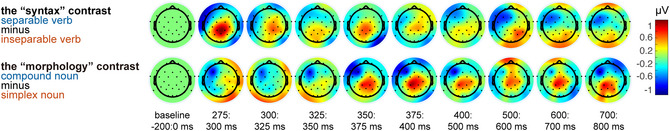
The scalp distribution for the baseline period (−200:0 ms), as well as for every 25 ms between 275:400 ms, and for every 100 ms between 400:800 ms. Here, we plot scalp distribution 275:400 ms in smaller (i.e., 25 ms) steps to be more comparable with the results of our topographical similarity analysis.

The significant interaction between complexity and ROI suggests that structural complexity indeed modulated neural responses in at least some regions of the scalp. In order to better understand the topography of the structural complexity effect and confirm that it was similar for both morphology and syntax, we followed up the significant interaction with a 2 (contrast type) × 2 (complexity) repeated‐measure ANOVA for each of the five ROIs separately. For the left anterior ROI (Fig. 2b,d), we indeed observed a significant main effect of complexity, *F*(1,23) = 7.47, *p* = .012; the main effect of contrast type and the interaction effect were not significant (*p*s > .19). For the centro‐parietal ROI (Fig. 2c,e), we also observed a significant main effect of complexity, *F*(1,23) = 6.83, *p* = .016, along with a main effect also for contrast type, *F*(1,23) = 6.14, *p* = .021, but not an interaction effect, *F*(1,23) = 1.12, *p* = .30. For the other three ROIs, no statistically significant effects were observed (*p*s > .14). In all, the ERP analyses seemed to suggest a left anterior effect accompanying a centro‐parietal effect in the LAN time window (275:400 ms), which is in line with visual inspection of the temporal evolution of the scalp distributions (Fig. 3).

One remaining question is whether differences in noun concreteness contributed to the effect observed in the morphology contrast for the noun conditions. Although the mean difference in concreteness ratings was quite small (Δ .19 on a 1–7 Likert scale), it was statistically reliable, and therefore it is possible that differences in concreteness rather than morphological status contributed to the difference between compound and simplex nouns. In order to evaluate this possibility, we conducted follow‐up tests leveraging individual differences in concreteness ratings (cf., Xu & Li, 2020). If the greater LAN for compound nouns were due to concreteness, we would expect the participants who rated the compound nouns as more concrete than the simplex nouns to demonstrate a more pronounced LAN effect relative to participants who rated the compound nouns as similarly concrete as (or even less concrete than) simplex nouns. This would predict a negative correlation with the LAN effect across participants. However, this correlation was not significant, and numerically went in the opposite direction of what would be predicted (Pearson's *r* = 0.32, *p* = .12). Therefore, we believe it is unlikely that the small difference in concreteness ratings contributed to the ERP differences observed in the compound noun–simplex noun contrast.

Additionally, as effects in the later P600 time window have been considered to reflect syntax‐related processes (cf., Afoian, Vulchanova, & Baggio, 2025), we also exploratorily analyzed this time window (400:700 ms): the effects were qualitatively the same as in the LAN time window (see Supporting Information). As effects in this time window did not come out in the following complementary topographical similarity analysis, we do not further discuss this result.

### 2.3 Topographical similarity analyses

**Fig. 4:**
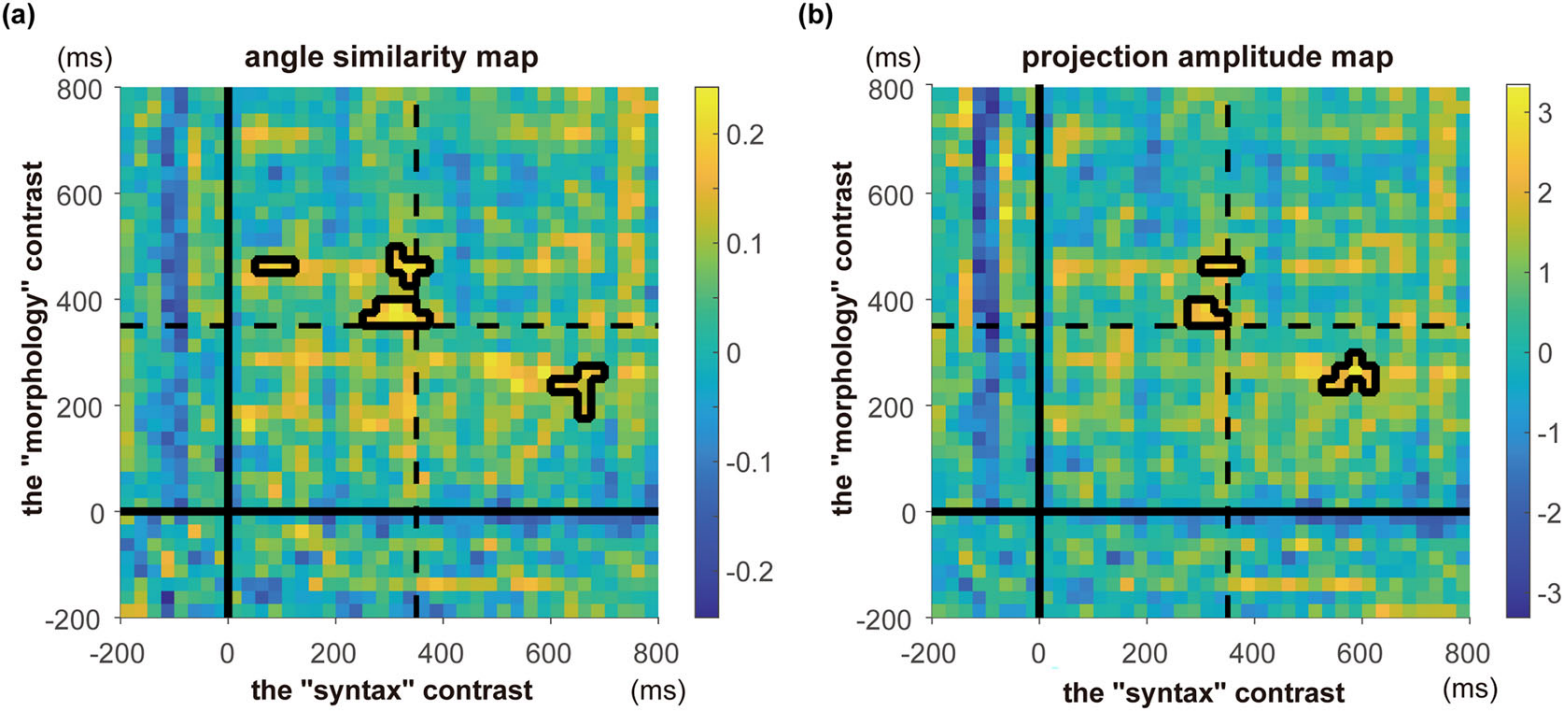
(a) Angle (cosine) similarity map from the topographical similarity analysis. The significant (*p* < .001, false discovery rate [FDR]‐corrected) clusters are outlined in black. The solid lines represent the onset of critical stimuli; the dashed lines indicate 350 ms post‐stimulus presentation. (b) Projection amplitude map from the topographical similarity analysis. The significant (*p* < .001, FDR‐corrected) clusters are outlined in black. The solid lines represent the onset of critical stimuli; the dashed lines indicate 350 ms post‐stimulus presentation.

The second component of our analyses was aimed at explicitly testing the topographical similarity of the “syntax” and “morphology” contrasts across time. This test identified at what points in time the scalp topographies for the two contrasts were similar, in increments of 25 ms (for details, see the Methods section). A significant cluster including time window t_1_ on the x‐axis (the time course of the syntax contrast) and time window t_2_ on the y‐axis (the time course of the morphology contrast) signifies that the scalp distribution in time window t_1_ for the syntax contrast resembles that in time window t_2_ for the morphology contrast. The analysis first identified potential clusters with a *p* < .05 threshold and retained only clusters that survived an α = 0.001 threshold with false discovery rate (FDR) correction. Significant clusters are marked with black contours in Fig. 4a,b, for the angle (cosine) similarity analysis and projection amplitude analysis, respectively. Four significant clusters were identified in the angle similarity analysis, spanning [50:125 ms (syntax), 450:475 ms (morphology)], [300:375 ms, 425:500 ms], [250:375 ms, 350:400 ms], and [600:700 ms, 175:275 ms] respectively. Here, [250:375 ms, 350:400 ms] refers to a cluster that extends 250:375 ms along the x‐axis (the time course for the “syntax” contrast) and extends 350:400 ms along the y‐axis (the time course for the “morphology” contrast), corresponding to the central cluster in Fig. 4a. Three significant clusters were identified in the projection amplitude analysis, spanning [300:375 ms, 450:475 ms], [275:350 ms, 300:400 ms], and [525:625 ms, 225:300 ms]. Critically, both analyses revealed a cluster at 350 ms across contrasts, in line with our LAN time window.

Interestingly, for the cluster around 350 ms, similar topographies appeared to emerge earlier for the syntax contrast than for the morphology contrast. This is in line with visual inspection of the scalp distributions around 350 ms (Fig. 3). Indeed, the exact time course for morpho‐syntactic structure building has been found to be affected by linguistic factors (Slaats, Meyer, & Martin, 2024).

## 3 Discussion

In the current study, we observed that both syntactic complexity and morphological complexity modulated left anterior and centro‐parietal electrodes in the same direction at 275:400 ms. The similarity of the neural response to the two contrasts was corroborated by topographical similarity analyses, which identified a cluster of similarity around ∼350ms. The similarity in the ERP responses for the morphological and syntactic contrasts is in line with the hypothesis that there is no qualitative difference between morphological and syntactic structure building (the “syntax‐all‐the‐way‐down” hypothesis; Krauska & Lau, 2023; Oseki & Marantz, 2020). As discussed below, these results could also be compatible with the hypothesis that morphological and syntactic structure are distinct but share common structure‐building routines.

### 3.1 Neural responses to separable verb compounds

In the current study, we observed left anterior and centro‐parietal effects for the “syntax” contrast, that is, between separable and inseparable verbs, in the 275:400 ms time window. To restate the linguistic fact, the separable verbs differ from inseparable verbs in that they are separable by other syntactic units, suggesting that separable verbs correspond to a more complex syntactic structure compared to inseparable verbs (see Section 1.1). Because both types of verbs are semantically non‐compositional and consist of two morphemes, these neural effects should be attributed to the inference of *syntactic* structure.

Several previous EEG studies have also examined Mandarin separable verbs (Gu, Yang, Ma, & Shen, 2018; Gu, Yu, & Ma, 2011; Zhang & Jiang, 2010) but with a different goal and a correspondingly different approach. As these studies assumed a lexicalist architecture, they asked whether separable verbs are “words” or “phrases” by comparing whether the ERP to separable verbs was more similar to words or to phrases. Zhang and Jiang (2010) found that separable verbs differed from words in many components (P200, N400, and P600) but from phrases only in P600; however, Gu et al. (2018) failed to observe a significant effect for the P600. These mixed results may have arisen due to additional factors in the materials these studies used for comparison; it is unclear whether all the items in the “word” conditions were verbs, and it is also unclear whether transitivity was matched across conditions. Furthermore, although aimed at distinguishing differences between “word” and “phrase” processing, these studies employed lexical decision or semantic categorization tasks, in which phrase comprehension is less likely to proceed normally. To our knowledge, no prior study has compared separable and inseparable verbs within sentence contexts while matching their transitivity, which we take to be crucial for evaluating their processing differences.

Since we used an average re‐reference rather than the mastoid re‐reference used by most language EEG studies, we need to be cautious about making assumptions about the relationship between the topography of our ERP effects and those observed in prior studies. Broadly speaking, however, our current left anterior and centro‐parietal effects in the LAN time window are in line with a previous ERP study, which observed a LAN effect in a manipulation of Dutch particle verbs (Piai, Meyer, Schreuder, & Bastiaansen, 2013; with averaged mastoid re‐reference). Dutch particle verbs consist of a particle and a head verb, which together can carry an idiomatic meaning, and as in the case of Mandarin separable verbs, they preserve this meaning even when they are syntactically separated.^3^ For example, the particle verb *voor‐spiegelen* (before‐mirror, “predict/promise”) can be separated, with the particle preceding the head verb:

(3) De   bank   spiegelt  haar  nierwste  klanten   hoge   winsten  voor.
 The  bank  mirrors   her   newest  customers  high  profit   before.
 “The bank promises high profits to its latest customers.”

Piai and colleagues compared the ERP responses for the head verbs that could appear in particle verbs against “inseparable” verbs that could not form a particle verb with any particle, when they were embedded in sentences. They hypothesized that, although the head verbs could also stand alone, participants would still consider the more complex particle‐verb syntactic structure already at the head verb (particularly since participants encountered many particle verbs in the experiment). Indeed, they observed a LAN effect in which the head verbs that could participate in particle verb constructions exhibited a more negative effect in left anterior electrodes between around 300–550 ms relative to the verbs that could not, consistent with the hypothesis that the more complex syntax of the particle verb construction put greater demands on working memory. One alternative explanation for this effect was that it could simply reflect greater syntactic uncertainty, although Piai et al. (2013) considered this explanation less likely since they failed to see a difference in the LAN response between head verbs that could take a wider range of particles versus a smaller range. The current study convergingly suggests that mere syntactic structure inference/building is enough to elicit a LAN‐like effect, as we presented both parts of intransitive separable verbs at once to the subjects, minimizing the role of syntactic prediction.

Although both Mandarin separable verbs and Dutch/German particle verbs are separable, there appear to be intriguing differences between the two phenomena, which would serve as a fertile ground for future research. Interestingly, for Mandarin separable verbs, the type of syntactic components that could go in between, as well as the syntactic operations that they could undergo (e.g., movement, reduplication), seem to be quite idiosyncratic. As an example, consider 帮忙 (bang1mang2, [help]‐[busy], “help”) and 费心 (fei4xin1, [cost]‐[heart], “take trouble”). Although they are both separable verbs, they exhibit different acceptability profiles when separated by certain syntactic units and when undergoing movement. While bang1mang2 can be separated in 帮了一个大忙 (bang1le0yi2ge0da4mang2, bang1 ASP one CL big mang2, “did a huge favor”), 费了一个大心 (fei4le0yi2ge0da4xin1, fei4 ASP one CL big xin1, intended “took huge trouble”) is unacceptable. While 忙他帮了 (mang2ta1bang1le0, mang2 he bang1 ASP, “he helped”) is fine, 心他费了 (xin1ta1fei4le0, xin1 he fei4 ASP, intended: “he took some trouble”) is tricky. This is just one pair of examples among many Mandarin separable verbs, many syntactic components that could go in between, and many syntactic operations that they could undergo (e.g., movement, reduplication of the first part; see Siewierska et al., 2010; Wang, 2011). This interesting idiosyncrasy remains to be documented comprehensively. How do native speakers come to learn this idiosyncrasy? Are there common factors that could account for the idiosyncrasies in Mandarin separable verbs and in German/Dutch particle verbs (see Schoenmakers & Foolen, 2022; Trotzke, Quaglia, & Wittenberg, 2015)? All these interesting questions await future research.

A separate question is where in the brain the ERP effect originates from and what that indicates about its functional interpretation. Many magnetoencephalography (MEG) studies have also tried to single out syntax (i.e., controlling for factors other than syntactic structure complexity) in order to examine brain responses sensitive to syntactic structures, and they have observed effects in the posterior temporal lobe (PTL) in somewhat similar time windows (Flick & Pylkkänen, 2020; Law & Pylkkänen, 2021; Matar et al., 2021). For example, Law and Pylkkänen (2021) examined MEG responses in the left PTL when a list was embedded in a sentence versus a longer list and observed a difference in the 333–389 ms time window. This is also in line with many functional magnetic resonance imaging (fMRI) studies supporting the involvement of the PTL in syntactic structure inference (Matchin, Hammerly, & Lau, 2017; Matchin & Hickok, 2020). We thus speculate that our ERP effects may originate from the PTL, which remains to be confirmed by future studies with techniques with a better spatial resolution (e.g., MEG and fMRI).

Although we have argued that our current ERP effect reflects sensitivity to syntactic structure, we should note that it is unclear if the effect reflects the recruitment of stored structured syntactic objects or “active” online structure building. The difference between these two processes is subtle but important. During syntactic structure building in language comprehension, one may consult structured objects (e.g., specific syntactic treelets) stored in long‐term memory, but ad hoc syntactic structure building is more than just “reinstantiating” all relevant stored objects. However, it is difficult to empirically disentangle the two processes as of now, because the neural implementation of syntactic structure building remains highly debated (Ding, 2023; Ding, Melloni, Zhang, Tian, & Poeppel, 2016; Krauska & Lau, 2023; Martin, 2020; Matchin & Hickok, 2020; Meyer, 2018; Murphy, 2020, 2024; Kazanina & Tavano, 2023; Pylkkänen, 2019), as well as the question of how long‐term linguistic objects are stored (Krauska & Lau, 2023; Murphy, 2024; Poeppel & Idsardi, 2022). Also note that on the syntax‐all‐the‐way‐down view, the notion of “morphological decomposition” can be understood as a part of morphosyntactic structure inference/structure‐building.

While the current results are consistent with the predictions of the syntax‐all‐the‐way‐down view, it is important to note that there are alternative theoretical perspectives that could also capture the commonality we observe across the “syntax” and “morphology” contrasts here without fully reducing syntax and morphology to the same data structure. For example, a theory like Construction Morphology (e.g., Booij, 2010) holds syntactic and morphological structures to be distinct but acknowledges similarities between those structures and thus could appeal to common processing routines acting to build those structures, in order to explain the kind of results observed here. Both explanations thus differ from traditional views in emphasizing commonalities between morphological and syntactic structure and the processes by which they are combined but make different commitments about the extent of those commonalities. Although developing psychological or neural measures that can dissociate details of data format are challenging, perhaps one direction for future work could be to explore priming manipulations across morphological and syntactic structures predicted to be identical on one account and distinct on the other.

### 3.2 Neural responses to Mandarin noun compounds versus simplex forms

Our left anterior and centro‐parietal effects for the “morphology” contrast (i.e., compound vs. simplex forms) in a sentence context can be seen as a replication of Y. Wei et al. (2023) earlier findings, with a similar time window. Y. Wei and colleagues employed a lexical decision task and observed a LAN effect in comparing Mandarin compounds and simplex forms with a variety of parts‐of‐speech. Our current study extends and strengthens their findings, as the results of isolated‐word paradigms do not always agree with the processing dynamics observed when the same items are embedded in sentential contexts (see Morris, 2017). Mirroring this point, a recent MEG study on auditory word processing revealed different neural substrates when words are processed in isolation versus in continuous speech (Gaston et al., 2023). Concerns have also been raised about the lack of naturalness of isolated‐word comprehension paradigms (Hamilton & Huth, 2020; Salmelin, Helenius, & Kuukka, 1999). Our corroborating evidence from a sentence paradigm is thus useful in demonstrating that the differential processing Y. Wei and colleagues observed for compound Mandarin forms in lexical decision generalizes to how they are processed in integrated sentence contexts.

A component that has been argued to be sensitive to morphological structure in the MEG literature is the M350 (Brooks & Cid de Garcia, 2015; Cavalli et al., 2016; Fiorentino & Poeppel, 2007; Hsu et al., 2019; Stockall & Marantz, 2006; for reviews, see Leminen, Smolka, Dunabeitia, & Pliatsikas, 2019; Royle & Steinhauer, 2023). The term “M350” sometimes refers to different measurements around 350 ms after the word onset, including the root mean square peak around 350 ms (Fiorentino & Poeppel, 2007), as well as neural responses in posterior temporal regions around 350 ms (Brooks & Cid de Garcia, 2015; Cavalli et al., 2016; Hsu et al., 2019). The left anterior and centro‐parietal effects observed in our current EEG study may reflect the same neural processes as M350, based on its time window. However, the neural source of the current effects remains to be confirmed by future MEG studies with better spatiotemporal resolution.

### 3.3 Similarity between “syntactic” and “morphological” effects

In short, in the current EEG study, we observed a similar LAN effect for both the “syntax” and the “morphology” contrasts. Within the a priori 275:400 ms time window, we observed similar left anterior and centro‐parietal effects that are both sensitive to syntactic structure complexity, but also morphological structure complexity, within the same group of subjects. This observation was corroborated by our topographical similarity analyses across the two contrasts, revealing a significant cluster around 350 ms, suggesting that the scalp distribution around 350 ms was similar across the two contrasts. The left anterior and centro‐parietal effects may originate from the same neural source, resulting in two opposing adjacent maxima on the scalp.

Form‐level visual parsing is considered to happen around 170 ms or earlier (Gwilliams, Lewis, & Marantz, 2016; Salmelin, 2007, 2010; Sigurdardottir, Ólafsdóttir, & Devillez, 2021; Tarkiainen, Helenius, Hansen, Cornelissen, & Salmelin, 1999). Based on the later time window of our effects (275:400 ms), it is thus likely to reflect abstract (i.e., non‐visual) morpho‐syntactic processes. In fact, even the ∼ 170 ms effects are already affected by morpho‐syntactic rules (Gwilliams & Marantz, 2018). From the traditional lexicalist point of view, the shared neural response across the “morphology” and “syntax” contrasts may come as a surprise; since a clear cut is assumed between morphological and syntactic rules and representations, the lexicalist view does not predict that they should share linguistic parsing processes, any more than phonology and syntax would be predicted to share linguistic parsing processes. On the other hand, shared process(es) are predicted by non‐lexicalist theories of language comprehension (for review, see Krauska & Lau, 2023; Oseki & Marantz, 2020), which hold that there is no qualitative difference between so‐called “syntactic” and “morphological” structure inference/building. In other words, syntax is “all the way down.”

Recent work closely related to construction grammar, known as Relational Morphology (Jackendoff & Audring, 2020a, 2020b), similarly eschews a strict syntax–lexicon distinction. That is, rather than language users having distinct modules for stored primitives and the procedures that can operate over them, all grammatical knowledge is in the form of declarative links between representations, stateable over “morphemes,” “words,” and phrases. These kinds of theories appear similarly compatible with the results we found here. Relational Morphology admits distinct morphosyntactic and phrasal syntactic levels of representation, though both rely on the same format of declared links between form‐syntax‐meaning. Language processing, then, under this framework, can be thought of as extended lexical access, where comprehension of some linguistic stimuli is considered to simply be the retrieval of the composite form‐meaning connections in long‐term memory, with different levels of representation interacting to converge on one or more meaning representations. This undifferentiated‐activation framework predicts that the retrieval and detection processes for morphosyntactic and phrasal syntactic objects are the same: activation spreading from sensory representations in morphophonology and phrasal phonology up to morphosyntax and phrasal syntax.

We note that it is possible that in other cases, different predictions could be made by construction grammar theories of processing and more procedural theories, where analyses are driven by transformations from one kind of representation to another, rather than the successful detection of an instantiation of a stored link between representations in a given stimulus. This is related to issues of online structure building versus reinstantiation of pieces of stored structure, whose effects are however in general difficult to dissociate, as mentioned previously.

Interestingly, in prior EEG and MEG work effects in this time‐window (LAN and M350) have been argued to reflect structure inference in the neuro‐morphology and the neuro‐syntax literature, respectively, and both have sometimes been suspected to originate from posterior temporal regions as reviewed above (note that although some studies suggest a left frontal source for LAN effects, many of those cases involved grammatical violations; e.g., Carreiras, Carr, Barber, & Hernandez, 2010; Friederici & Kotz, 2003). Our current study verifies that a similar effect for “morphology” and “syntax” can be observed within the *same* group of participants. Of course, a similar topography does not guarantee the exact same neural processes, yet it provides encouraging *prima facie* evidence awaiting future studies with better spatial resolution to confirm.

Indeed, either “morphology” or “syntax” may consist of a variety of different operations that may correspond to non‐identical neural signatures. Nevertheless, some neural signatures could be shared across different combinatorics, which may be governed by, for example, the topology of the morpho‐syntactic trees (e.g., those relevant to the depth or node count of the trees, see Brennan, Stabler, Van Wagenen, Luh, & Hale, 2016; Li, Brennan, Mahar, & Hale, 2016; Ohta & Sakai, 2017). These shared neural signatures (suggesting shared neural processes) may be the reason why we observed a similar neural response across the “morphology” and “syntax” contrasts in our current study, regardless of the idiosyncrasy in morpho‐syntactic combinatorics.

### 3.4 The opacity conundrum

One question is the degree to which effects like the current ones are modulated by semantic opacity: how much the meaning of the whole is intuitively related to the meaning of the parts. First, we would like to point out that Y. Wei et al. (2023) did not observe a significant modulation effect of opacity on LAN or N400 with Chinese compound nouns of different opacity. Although semantic opacity may still be an interesting factor to explore in the future, we would also like to point out some difficult theoretical challenges regarding “semantic opacity,” which we term “the opacity conundrum.”

To begin with, it is worth noting that there is no independent measurement of *semantic* opacity apart from subjective ratings. For one thing, it is hard, if not impossible, to prove that the measured ratings are purely of a semantic nature but not of a morpho‐syntactic nature at all (*the semantic purity problem*). For another, there is no obvious way to tell if the measured opacity is just a reflection or a natural result of different underlying morpho‐syntactic structures or that opacity affects morpho‐syntactic parsing in turn (*the direction of causality problem*). Another challenge for the opacity for complex nouns is that, opacity has to be defined somehow by the relations between the meaning of the whole linguistic expression and *the meaning of a morpheme*, which is an extremely ill‐defined concept in linguistics (*the morpheme meaning problem*). Notably, expert linguists’ judgments do not match perfectly with novice ratings (Gagné, Spalding, & Nisbet, 2016); in this case, it is hard to argue for one judgment over the other precisely because of a lack of independent measurement and easily operationalizable definition for semantic opacity (same for the construct “semantic transparency”). Therefore, although we acknowledge that the interplay between “opacity” and morpho‐syntactic parsing is an interesting research question, there are some deep theoretical problems about “opacity,” which need to be addressed in future research, namely, the semantic purity problem, the direction of causality problem, and the morpheme meaning problem.

That said, participants may have psychologically real “folk” theories—whether explicit or implicit—about the semantic meaning of the parts and their relations to the whole, and these folk theories may affect online processing of these linguistic expressions (Libben, 1998, 2003; Myers, 2006; Tsang, Zou, & Tse, 2022). Indeed, relevant semantic processes may drive a LAN effect in some other languages (Güemes, Gattei, & Wainselboim, 2019; Koester, Gunter, & Wagner, 2007), although semantic opacity did not drive a LAN effect in the study of Y. Wei et al. (2023) with Mandarin Chinese. This thus provides an interesting ground for future research.

Yet another factor on which compounds can vary is properties of the compound constituents themselves, such as constituent neighborhood size, which as a reviewer points out, has been shown to influence both behavioral and neurological responses (e.g., Huang et al., 2006; Lee et al., 2004; Li, Lin, Chou, Yang, & Wu, 2015; Tse, Yap, & Chan, 2024). Although we did not examine neighborhood size in the current study, we agree that examining the interaction between sentence context and constituent neighborhood size will be an important direction for future work.

### 3.5 The challenge of discerning morphological structures

Another question is whether our contrast between separable and inseparable verbs could be framed in terms of distinct morphological structures instead of varying syntactic complexity—for example, if separable verbs in fact do have a verb+object (VO) morphological structure, while the inseparable verbs have a different morphological structure. This would provide a potential alternative account for the current work, which preserves a “lexicalist” framework in which syntactic combination and morphological combination are distinct.

This possibility is worth considering, but it is famously challenging to distinguish these accounts. Taking the example of 跑步 (pao3bu4, [run]‐[step], “run”), the fact that “run” is a verb when used in isolation and “step” is a noun when used in isolation cannot be considered a priori evidence that the forms are associated with the same morpho‐syntactic category in the separable verb that contains them (Chen, 2015, Ch. 7.1), nor is it straightforward to rely on informal intuitions from speakers. Even if a consensus were reached about which compounds have which structures, it will remain difficult to empirically distinguish between theories in which these structures are syntactic versus morphological. Therefore, much more work will likely be needed to distinguish these two possible accounts. We believe these are important questions that future neurophysiological research on compound processing should continue to pursue.

Indeed, a number of researchers have assumed and/or have provided analytical arguments in favor of different morphological structures across Chinese compounds (Li, 1990; Li & Thompson, 1981; Liao, 2014; Myers, 2007; Packard, 2000), and experimental reports have also reported differences between compounds of different hypothesized structure types (Chung, Tong, Liu, McBride‐Chang, & Meng, 2010; Cui et al., 2018; Gao et al., 2022; Güemes et al., 2022; Hsu et al., 2019; Liu & McBride‐Chang, 2010; Sun et al., 2023), following the lexicalist tradition. We therefore also provide a morpho‐syntactic analysis of our stimuli following this tradition and count the occurrences of different structures: separable verbs (26 VO, 3 VV, 1 VAdj), inseparable verbs (11 VV, 9 VO, 5 AdvV, 3 AdjV, 2 NV), compound nouns (20 NN, 7 AdjN, 1 AdjV, 1 VN, 1 VO), and simplex nouns (30 monomorphemic). For item‐by‐item analysis, see Supporting Information. There appeared to be more VO structures in separable verbs, compared to inseparable verbs, and more VV structures in inseparable verbs, compared to separable verbs. Although prior EEG/ERP studies have identified reliable neural signatures for the complexity of morpho‐syntactic structures (e.g., across compounds and monomorphemic words, Lehtonen et al., 2007; Y. Wei et al. 2023), we are not aware of existing studies demonstrating a reliable modulation of ERPs by different structural types, which would be an interesting and important topic of future research. Notably, a same‐structure priming effect—for example, coordinative nouns/verbs priming coordinative nouns/verbs, compared to VO nouns/verbs priming coordinative nouns/verbs—in the P250 time window (220–300 ms) was observed in Chung et al. (2010). However, direct evidence for an ERP difference between VV and VO structures remains to be established. In particular, priming paradigms tend to be more sensitive than paradigms without primes, as priming paradigms capture neural activities that may evade the common averaging pipeline in other paradigms (Glezer, Jiang, & Riesenhuber, 2009; Grill‐Spector & Malach, 2001). Moreover, if the LAN were driven strictly by an internal structure contrast between VO and VV, one would not expect to observe it in the compound versus simplex noun condition, which consists mostly of NN and monomorphemic structures. Therefore, while acknowledging these structural variations, we consider it unlikely that the difference observed across conditions could be attributed to this lexical category variation rather than the complexity of the structures themselves. We believe that further investigation into the morphosyntactic analysis of these structures, as well as their psycholinguistic reflexes, will generate interesting and informative findings in the future, bridging analytical and experimental traditions.

Following this line, this discussion has important implications for future studies in stimuli construction: directly addressing this concern would require a matched and sufficient (at least 30) number of VO structures that are separable and inseparable verbs, such that this difference is only syntactic under the lexicalist tradition. Among our current inseparable verbs, there were only nine of the VO structure, and we found it challenging to find many more intransitive inseparable verbs of the VO structure. Future studies could either increase statistical power by largely increasing the sample size, or exploring comparable contrasts in other languages. Our current study thus serves as an initial stepping‐stone toward this direction.

## 4 Conclusion

In the current study, we observed a similar modulation of syntactic and morphological structure complexity on left anterior and centro‐parietal electrodes in a time window informed by prior research (275:400 ms). These qualitatively similar responses are in line with the predictions of non‐lexicalist theories of language comprehension, which propose that there is no qualitative difference across the neuro‐cognitive operations/computations involved in syntactic and “morphological” structure inference and building.

## 5 Methods

### 5.1 Subjects

Subjects were recruited and tested in Shanghai, China, and all of them self‐identified as native speakers of Mandarin Chinese. Subjects received monetary reimbursement for their participation. Written consent was acquired from each subject, and the procedures were approved by the Institutional Review Board of the University of Maryland and New York University Shanghai. Two subjects’ data were excluded from data analysis because of artifacts (e.g., blinking, drifts) in excessive (≥ 50%) epochs. The remaining 24 subjects (16 female) had an age of 19–29 (*M* = 23). One of the analyzed subjects was left‐handed, and all others were right‐handed, as measured by a translated version of the Edinburgh Handedness Inventory (Oldfield, 1971).

### 5.2 Stimuli

We first selected 30 items for each of the four compound types tested in this study: compound nouns, simplex nouns, separable verbs, and inseparable verbs. The compound and simplex nouns were disyllabic, matched in log10 bigram frequency in two large corpora (BCC multi‐domain corpus, Xun, Rao, Xiao, & Zang, 2016; simplex: 3.6, compound: 3.7, independent sample *t* test *p* = .33, two‐tailed; CCL2024 corpus, Zhan, Guo, Chang, Chen, & Chen, 2019; simplex: 3.9, compound: 3.9, independent sample *t* test *p* = .88, two‐tailed), as well as the stroke numbers for each character (1st character, simplex: 8.1, compound: 8.3, Mann–Whitney U test *p* = 1.0, two‐tailed; 2nd character, simplex: 6.9, compound: 7.7, independent sample *t* test *p* = .29, two‐tailed; the Mann–Whitney U test was administered instead of the independent sample *t* test whenever at least one compared distribution did not pass the Shapiro–Wilk test, *p* < .05). The simplex nouns were transliteration‐based loan words. Although a number of these loan words in Chinese are composed of two visually similar characters, for example, 咖啡 (ka1fei1, “coffee”; the two characters share the same radical to the left), 芭蕾 (ba1lei2, “ballet”; the two characters share the same radical on top), we were careful to exclude such items from our stimuli set. The separable and inseparable verbs were intransitive, disyllabic, and matched in log10 bigram frequency in two large corpora (BCC multi‐domain corpus; separable: 3.9, inseparable: 3.9, independent sample *t* test *p* = .92, two‐tailed; CCL2024 corpus; separable: 4.2, inseparable: 4.1, Mann–Whitney U test *p* = .23, two‐tailed), as well as the stroke numbers for each character (1st character, separable: 8.6, inseparable: 8.2, independent sample *t* test *p* = .55, two‐tailed; 2nd character, separable: 7.9, inseparable: 8.6, independent sample *t* test *p* = .36, two‐tailed). All compounds used in the study were selected to be intuitively non‐compositional in meaning, in that the meaning did not follow straightforwardly from the meaning of the parts, for example, for the compound 热狗 (re4gou3, hot‐dog, “hotdog”), its meaning is not a straightforward composition of the meaning of 热 (re4, “hot”) and 狗 (gou3, “dog”).

We then created a list of 120 sentences that contained the items from the four 30‐word compound sets. The idea was to embed the compound words in a low‐prediction neutral context. The sentential patterns and example sentences are illustrated in Table 2; for a comprehensive list of our stimuli see our open data. Specifically, for the 30 separable and inseparable verb sentences, 1/3 followed “wants to,” “does not want to,” and “really wants to.” In order to control for the semantic congruity of “wants to/does not want to/really wants to + verb” (which all consist of four characters) across the two conditions, we matched the 4‐gram frequency in two large corpora (cf., Lau et al., 2016). Because many of the 4‐grams did not appear in the corpus (despite them being large of their kind), we calculated log10(frequency+1) as log10 frequency and employed a Mann–Whitney U test; the two conditions were balanced in 4‐gram frequency (BCC multi‐domain corpus: separable: 0.39, inseparable: 0.34, *p* = .58; CCL2024 corpus: separable: 0.38, inseparable: 0.43, *p* = .77). Two balanced lists of names were created for the separable and inseparable verb sentences, and the names were counterbalanced across the 24 analyzed subjects. For separable and inseparable verb sentences, a comma always followed the verbs in order to ensure an intransitive reading because of an increasing trend in Mandarin where intransitive verbs can be somewhat acceptable with transitive usages (Liao & Tsai, 2023). An additional 60 filler sentences were adapted from another study (Liao & Lau, 2020), rendering 180 sentences for each subject.

### 5.3 Procedure

Subjects sat ∼60 cm in front of the screen. A total of 180 trials were run for each subject (30 trials for each of the four conditions + 60 filler sentences), with a different sentence presented in each trial. Each trial began with a fixation cross of 1000 ms, followed by presentation of the segments of this sentence in a segment‐by‐segment (i.e., RSVP, rapid serial visual presentation) manner, with a presentation duration of 600 ms for each segment, and a 200 ms interval between segments, for a total 800 ms stimulus‐onset asynchrony (Fig. 1b). The segments were presented in white characters against a black background, in 60 pt, Yahei font; they were presented at the center of the screen. In 1/3 of the trials, the last segment of the sentence (i.e., the continuation) was colored in green, and the subjects were instructed to respond to whether this continuation was congruent in this sentence, by pressing m (incongruent) and n (congruent) on the keyboard with their right hand (the responses did not have a time limit). In the other 2/3 of the trials, the last segment was presented on the screen in white briefly for 1000 ms, and subjects did not need to respond. The inter‐trial intervals were self‐paced; subjects pressed the space bar when they were ready to start the next trial.

After the experiment, the subjects were administered two post‐tests. The first, “separability” post‐test, aimed at testing their subjective rating of the separability of the 60 separable and inseparable verbs used in the experiment. Subjects rated the acceptability of the separable and inseparable verbs on a 1–7 Likert scale, when they were separated by aspect markers “了” (le0) or “过” (guo0). The aspect marker used for each verb in the post‐test was balanced across subjects; for example, half of the subjects were asked to rate the acceptability of “造了反” ([rebel‐] le0 [‐rebel]), and the other half of the subjects were asked to rate the acceptability of “造过反” ([rebel‐] guo0 [‐rebel]). Separability by aspect markers is a common and signature property for separable verbs (Siewierska et al., 2010; Wang, 2011). The second post‐test asked subjects to rate the concreteness of the 60 compound and simplex nouns used in the experiment on a 1–7 Likert scale, with an instruction adapted from prior concreteness studies in Chinese (Xu & Li, 2020; Yao, Wu, Zhang, & Wang, 2017).

### 5.4 Behavioral data analysis

Accuracy for the main experiment: Subjects’ responses to the continuation judgment task were recorded, and we calculated the accuracy for each subject.

Post‐test ratings: For the separability and concreteness ratings, we conducted pairwise *t* tests on the mean ratings for each subject by condition.

### 5.5 EEG recording

The EEG was recorded using a 32‐channel (Fp1, Fz, F3, F7, FT9, FC5, FC1, C3, T7, TP9, CP5, CP1, Pz, P3, P7, O1, Oz, O2, P4, P8, TP10, CP6, CP2, C4, T8, FT10, FC6, FC2, F4, F8, Fp2, Cz) active electrode system (Brain Vision actiCHamp; Brain Products) with a 1000 Hz sampling rate in an electromagnetically shielded and sound‐proof room. Electrodes were placed on an EasyCap, on which electrode holders were arranged according to the 10–20 international electrode system. The impedance of each electrode was kept below 25 kΩ. The data were referenced online to electrode Cz. Two additional EOG electrodes (HEOG and VEOG) were attached for monitoring ocular activity. The EEG data were acquired with Brain Vision PyCoder software and filtered online between DC and 200 Hz with a notch filter at 50 Hz.

### 5.6 EEG data analysis

EEG data were analyzed with EEGLAB and ERPLAB, along with customized MATLAB scripts. The EEG data were first subject to a 0.01 to 40 Hz band‐pass filter. Then bad channels were interpolated (spherical, 0–2 bad channels per subject), and the data were re‐referenced to the average reference across scalp electrodes. An average re‐reference was used because we did not have mastoid channels in our EEG setup. Then, −200:800 ms epochs were extracted and baselined to the mean response between −200:0 ms. Epochs with artifacts were identified and excluded with a simple voltage threshold of −100 to 100 µV. We then reviewed the data and, if needed, adjusted the voltage threshold for individual subjects. A few trials were excluded due to a technical error that resulted in an inaccurate presentation duration. Then the ERPs for each condition were calculated within each subject. The number of trials left for each condition after artifact rejection is as follows: simplex nouns (26.3 trials, range 18–30), compound nouns (26.7 trials, range 21–30), separable verbs (27.3 trials, range 19–30), inseparable verbs (27.0 trials, range 19–30).

#### 5.6.1 ERP analysis

We conducted an omnibus repeated‐measure ANOVA analysis, with ROI (five ROIs, Fig. 1c), contrast type (the morphology contrast, the syntax contrast), and complexity (more complex, less complex morpho/syntactic structure) as independent variables and the mean response within the 275:400 ms (Y. Wei et al., 2023) time window for each subject in each ROI as the dependent variable (thus, a 5 × 2 × 2 three‐way ANOVA). Greenhouse–Geisser correction was applied whenever sphericity was violated. The ANOVA analyses were conducted in JASP 0.18.1 (JASP Team, 2023).

#### 5.6.2 Topographical similarity analysis

Topographical similarity analysis (i.e., representational similarity analysis, spatial similarity analysis) is an analysis for comparing the topographical distribution of ERP responses that is free from bias in electrode selection (Murray, Brunet, & Michel, 2008; Tian & Huber, 2008; Tian et al., 2011; Wang, Zhu, & Tian, 2019). Quantifying topographical similarity with this method has been used to hallmark similarity in the underlying neural processes in many language comprehension studies (Ding, Ten Oever, & Martin, 2024; Huang, Feng, & Qu, 2023; Hubbard & Federmeier, 2021; Yang, Cai, & Tian, 2020; Wang & Kuperberg, 2023; Wang et al., 2020; Wei, Huang, Feng, and Qu, 2023; Liu, Fan, Chen, & Zhao, 2023).

For a more stable topography, we first averaged the ERP responses every 25 ms for each subject within the −200:800 ms epochs, collapsing 1000 time points (−200:800 ms) to 50 time points. Then we obtained the scalp topography for the “morphology” contrast (compound minus simplex nouns) and the “syntax” contrast (separable minus inseparable verbs) for each subject. Then, we calculated the angle (cosine) similarity and projection amplitude (Tian & Huber, 2008; Tian et al., 2010) across the two contrasts at each of the 25 ms time windows. We treated the ERP amplitudes of each scalp electrode (31 in total) as a unique dimension of a vector. Suppose the ERP amplitudes for the “morphology” contrast at time window i forms a 1 × 31 vector **A_i_**, and the ERP amplitudes for the “syntax” contrast at time window j forms a 1 × 31 vector **B_j_**, the angle similarity of the two vectors was calculated as

$$\mathrm{AngleSimilarity}\left(i,j\right)=\frac{A_{i}B_{j}}{\left|A_{i}\right|\left|B_{j}\right|}$$

And the projection amplitude of the two vectors was calculated as the mean of the projection of vector **A_i_** on **B_j_** and the projection of vector **B_j_** on **A_i_**:

$$\mathrm{ProjectionAmplitude}\left(i,j\right)=\left(\left|\frac{A_{i}B_{j}}{B_{j}B_{j}}B_{j}\right|+\left|\frac{B_{j}A_{i}}{A_{i}A_{i}}A_{i}\right|\right)\mathrm{sign}\left(\mathrm{Angl}\mathrm{eSim}\mathrm{ilar}\mathrm{ity}\left(i,j\right)\right)/2$$

We calculated the angle similarity and projection amplitude for every time window of the “morphology” contrast and each time window of the “syntax” contrast. Thus, we obtained a 50 × 50 map for angle similarity and projection amplitude for each subject. First, at each time window (i,j) in the angle similarity map (which contains one single angle similarity value for each subject), we conducted a one‐sample *t* test against 0 (that the two topographies are orthogonal to each other, i.e., not similar at all), *p* < .05 (uncorrected). Then a set of potential clusters was identified based on the temporal adjacency of these significant time windows. After that, for each potential cluster, we calculated the mean within it for each subject and compared this distribution against 0 (i.e., no similarity) with a one‐sample *t* test, rendering a new *p*‐value for each temporal cluster. Only the clusters that survived an α = 0.001 threshold (FDR‐corrected with the fdr_bh() function, Benjamini & Hochberg, 1995; Groppe, 2023) are outlined in Fig. 1c. A similar analysis was run for the projection amplitude map as well (Fig. 1d).

## Funding information

This work is supported by NSF #1749407 (to E.L.) and NSF Graduate Research Fellowship under Grant No. DGE 1840340 (to S.M.).

## Conflict of interest

The authors declare no conflict of interest.

## Supporting information



Supporting Information

## Data Availability

The stimuli, data, and data processing scripts are publicly shared on OSF: https://osf.io/qt6x8/.

## Notes

1 Note that the analogy is inexact as English does not have verb–verb compounds; this is only meant to give an intuition.

2 By morphological or syntactic “representations,” we refer to the mental representations for the morpho‐syntactic nodes and the relations between/among each other in morpho‐syntactic trees.

3 Idiomatic phrasal verbs in English (e.g., “heat up”; Cappelle, Shtyrov, & Pulvermüller, 2010; Paulmann, Ghareeb‐Ali, & Felser, 2015) might be somewhat similar as well.
