# Supplementary material for: Shared Neural Computations for Syntactic and Morphological Structures: Evidence From Mandarin Chinese
Source: Cogn Sci. 2026 May 12;50:e70220. doi: 10.1111/cogs.70220 (PMC13167000; doi:10.1111/cogs.70220)
Supplement: Supplementary file 1 — Supporting Information [file COGS-50-e70220-s001.pdf]

Supplementary Materials

Morpho-syntactic analysis for our stimuli

Here we provide a morpho-syntactic analysis for our stimuli following classic works on lexicalist morphology (Li & Thompson, 1981; Packard. 2000; Liao, 2014). There are, however, cases where this traditional morpho-syntactic analysis may lead to uncertain outcomes (see e.g., the 跑步 pao3bu4 "run" case in the main text). In this section, we would make the closest judgement (e.g., classifying 跑步 pao3bu4 as VO regardless). One can relatively safely label this case as VN, but we keep the notation of "VO" here per convention.

**condition**	**expression**	**meaning**	**Morpho-syntactic analysis**
separable	帮忙	help	VO
separable	结婚	get.married	VO
separable	吃亏	be.at.disadvantage	VO
separable	费心	take.the.trouble	VO
separable	执勤	be.on.duty	VO
separable	请假	ask.for.leave	VO
separable	开课	begin.class	VO
separable	请客	invite.for.meal	VO
separable	鼓掌	applause	VO
separable	理发	have.haircut	VO
separable	丢人	lose.face	VO
separable	告状	tell.on	VO
separable	搞鬼	make.mischief	VO
separable	带头	take.the.lead	VO
separable	起床	get.up	VO
separable	吹牛	brag	VO
separable	捣乱	make.trouble	VO
separable	出面	step.in	VO
separable	起哄	jeer	VO
separable	让步	compromise	VO
separable	说情	speak.for	VO
separable	住院	in.hospital	VO
separable	罢工	go.on.strike	VO
separable	宣誓	make.an.oath	VO
separable	打猎	hunt	VV
separable	造反	rebel	VAdj
separable	跑步	run	VO

separable	退休	retire	VV
separable	剪彩	cut.the.ribbon	VO
separable	行贿	bribe	VV
inseparable	逃跑	escape	VV
inseparable	修行	practice Buddhism or Taoism	VO
inseparable	怒吼	shout.in.rage	AdjV
inseparable	应酬	take.part.in.social.activities	VO
inseparable	休息	rest	VV
inseparable	乔装	disguise	VO
inseparable	爬行	crawl	VV
inseparable	迟疑	hesitate	AdjV
inseparable	潜逃	abscond	AdvV
inseparable	与会	participate.in.meeting	VO
inseparable	独处	live.alone	AdvV
inseparable	奋斗	work.hard	VV
inseparable	成功	succeed	VO
inseparable	悔过	regret	VO
inseparable	痛哭	cry.hard	AdvV
inseparable	失踪	go.missing	VO
inseparable	社交	socialize	NV
inseparable	自保	protect.oneself	NV
inseparable	恐慌	be.panic	VV
inseparable	合作	cooperate	AdvV
inseparable	贿选	bribe.in.election	VV
inseparable	失控	lose.control	VO
inseparable	远足	hike	AdjV
inseparable	漂泊	have.a.wandering.life	VV
inseparable	游荡	wander	VV
inseparable	屈服	give.in	VV
inseparable	失败	fail	VV
inseparable	败北	fail	VO?
inseparable	叛逆	be.rebellious	VV
inseparable	窃笑	laugh.secretly	AdvV

**condition**	**expression**	**meaning**	**Morpho-syntactic analysis**
compound	讲座	lecture	VN
compound	火鸡	turkey	NN
compound	水仙	narcissus	NN

<table>
<tr><td>compound</td><td>黑板</td><td>blackboard</td><td>AdjN</td></tr>
<tr><td>compound</td><td>钢笔</td><td>fountain pen</td><td>NN</td></tr>
<tr><td>compound</td><td>雪糕</td><td>ice cream</td><td>NN</td></tr>
<tr><td>compound</td><td>大衣</td><td>overcoat</td><td>AdjN</td></tr>
<tr><td>compound</td><td>银耳</td><td>white woodear</td><td>NN</td></tr>
<tr><td>compound</td><td>司仪</td><td>host</td><td>VO</td></tr>
<tr><td>compound</td><td>洋葱</td><td>onion</td><td>NN</td></tr>
<tr><td>compound</td><td>月饼</td><td>mooncake</td><td>NN</td></tr>
<tr><td>compound</td><td>酸奶</td><td>yogurt</td><td>AdjN</td></tr>
<tr><td>compound</td><td>电池</td><td>battery</td><td>NN</td></tr>
<tr><td>compound</td><td>钢琴</td><td>piano</td><td>NN</td></tr>
<tr><td>compound</td><td>网球</td><td>badminton</td><td>NN</td></tr>
<tr><td>compound</td><td>蓝牙</td><td>bluetooth</td><td>AdjN</td></tr>
<tr><td>compound</td><td>贴吧</td><td>bbs</td><td>NN</td></tr>
<tr><td>compound</td><td>金鱼</td><td>goldfish</td><td>NN</td></tr>
<tr><td>compound</td><td>银杏</td><td>ginkgo</td><td>NN</td></tr>
<tr><td>compound</td><td>燕麦</td><td>oat</td><td>NN</td></tr>
<tr><td>compound</td><td>热狗</td><td>hotdog</td><td>AdjN</td></tr>
<tr><td>compound</td><td>舞会</td><td>dance party</td><td>NN</td></tr>
<tr><td>compound</td><td>茶几</td><td>coffee table</td><td>NN</td></tr>
<tr><td>compound</td><td>小吃</td><td>snack</td><td>AdjV</td></tr>
<tr><td>compound</td><td>白菜</td><td>Napa cabbage</td><td>AdjN</td></tr>
<tr><td>compound</td><td>龙眼</td><td>longan fruit</td><td>NN</td></tr>
<tr><td>compound</td><td>冰柜</td><td>freezer</td><td>NN</td></tr>
<tr><td>compound</td><td>宽带</td><td>broadband</td><td>AdjN</td></tr>
<tr><td>compound</td><td>煤气</td><td>gas</td><td>NN</td></tr>
<tr><td>compound</td><td>龙舟</td><td>dragon boat</td><td>NN</td></tr>
<tr><td>simplex</td><td>沙龙</td><td>salon</td><td rowspan="11">simplex</td></tr>
<tr><td>simplex</td><td>培根</td><td>bacon</td></tr>
<tr><td>simplex</td><td>马达</td><td>motor</td></tr>
<tr><td>simplex</td><td>摩托</td><td>motorbike</td></tr>
<tr><td>simplex</td><td>探戈</td><td>tango</td></tr>
<tr><td>simplex</td><td>曲奇</td><td>cookie</td></tr>
<tr><td>simplex</td><td>夹克</td><td>jacket</td></tr>
<tr><td>simplex</td><td>芝士</td><td>cheese</td></tr>
<tr><td>simplex</td><td>模特</td><td>model</td></tr>
<tr><td>simplex</td><td>吐司</td><td>toast</td></tr>
<tr><td>simplex</td><td>披萨</td><td>pizza</td></tr>
</table>

simplex	可乐	cola	simplex
simplex	巴士	bus	
simplex	吉他	guitar	
simplex	坦克	tank	
simplex	圣代	sundae	
simplex	博客	blog	
simplex	布丁	pudding	
simplex	扑克	poker	
simplex	沙拉	salad	
simplex	汉堡	hamburger	
simplex	派对	party	
simplex	沙发	sofa	
simplex	桑巴	samba	
simplex	摩卡	mocha	
simplex	拿铁	latte	
simplex	雪茄	cigar	
simplex	雷达	radar	
simplex	桑拿	sauna	
simplex	引擎	engine	

We summarize the occurrences of difference structures with the following table:

	**counts**
**separable verbs**	26 VO, 3 VV, 1 VAdj
**inseparable verbs**	11 VV, 9 VO, 5 AdvV, 3 AdjV, 2 NV
**compound nouns**	20 NN, 7 AdjN, 1 AdjV, 1 VN, 1 VO
**simplex nouns**	30 monomorphemic

Supplementary Figures

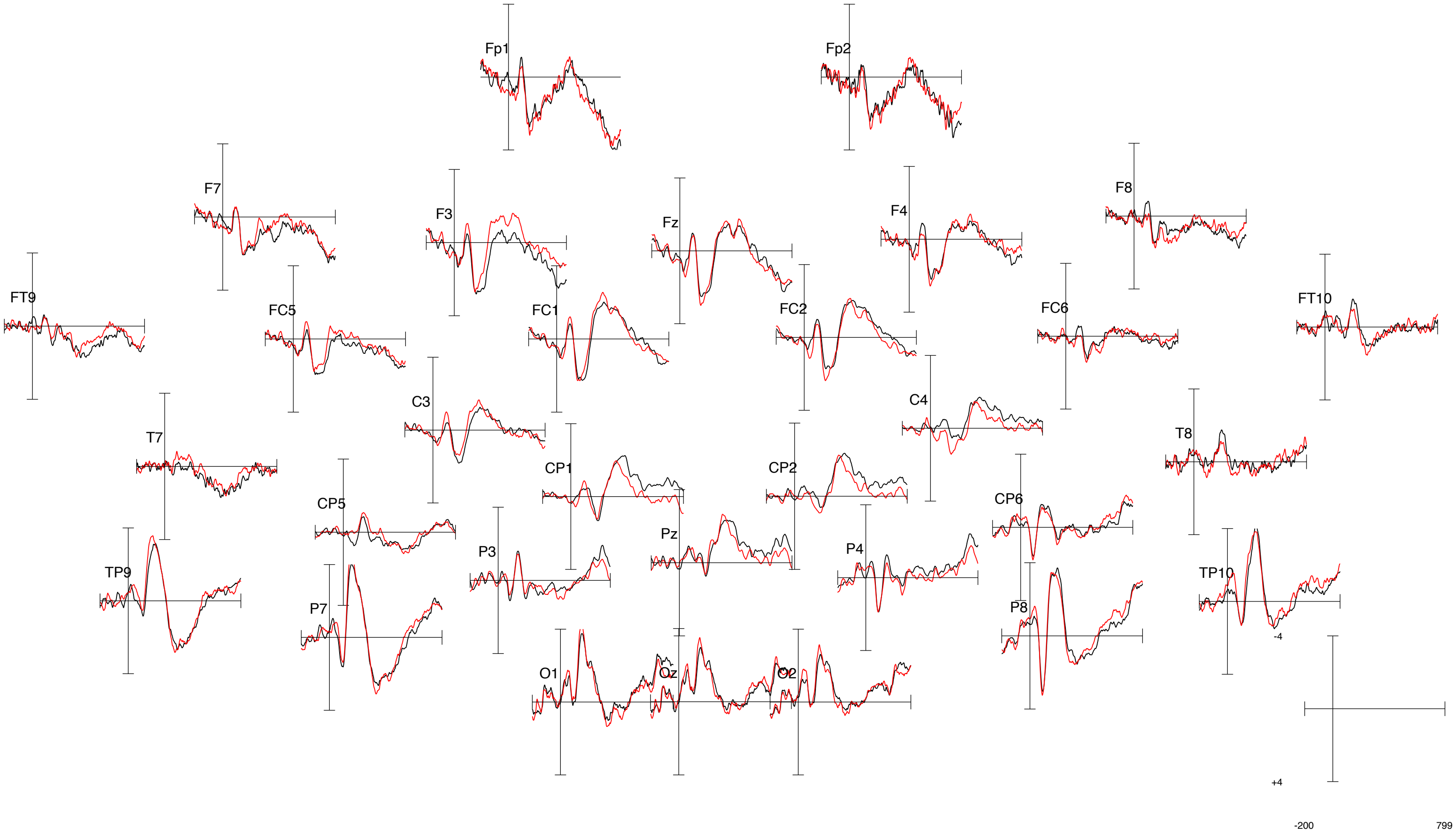

Figure S1. ERP waveforms of all scalp electrodes for the “morphology” contrast. Red: compound nouns; black: simplex nouns.

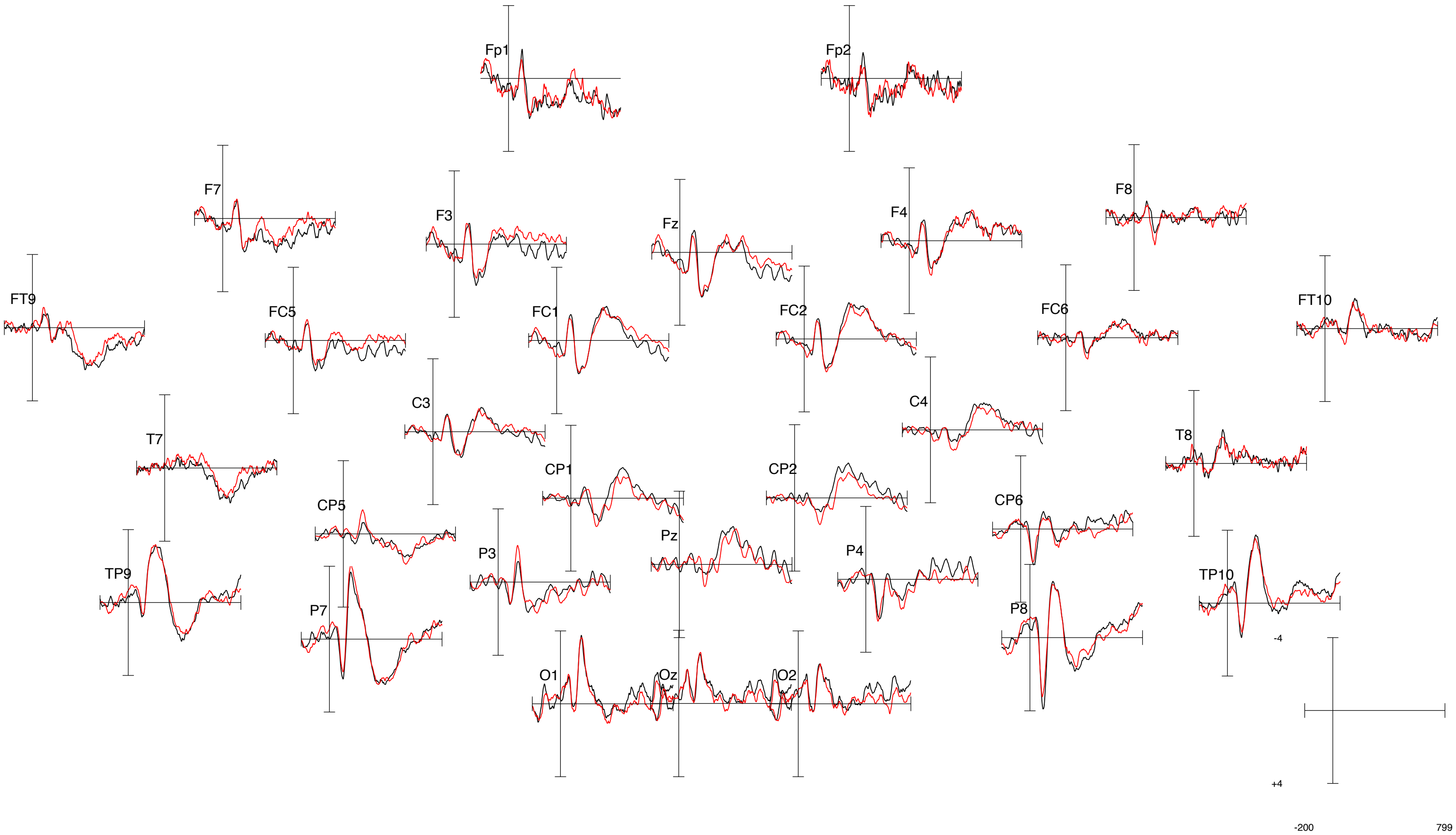

Figure S2. ERP waveforms of all scalp electrodes for the “syntax” contrast. Red: separable verbs; black: inseparable verbs.

Supplementary analysis

Omnibus ANOVA analysis (400:700 ms). Similarly, we conducted a repeated measures ANOVA analysis, with ROI (5 ROIs, Figure 2a), contrast type (the morphology contrast, the syntax contrast), and complexity (more complex, simpler morpho/syntactic structure) as independent variables, and the mean response within the 400:700 ms time window in each ROI as the dependent variable. Greenhouse-Geisser correction was applied whenever sphericity was violated.

The three-way ANOVA again revealed a significant main effect of ROI, $F(2.88,66.25) = 5.36$, $p = 0.003$, as well as an interaction effect between complexity and ROI, $F(2.54,58.50) = 3.37$, $p = 0.031$; all other effects were not statistically significant (p's > 0.22).

Based on the interaction effect between complexity and ROI, we then conducted a 2 (contrast type) × 2 (complexity) ANOVA for each of the five ROIs separately. For the LAN ROI, we also observed a significant main effect of complexity, $F(1,23) = 8.99$, $p = 0.006$; the main effect of contrast type and the interaction effect were not significant (p's > 0.46). For the central-parietal ROI, we also observed a significant main effect of complexity, $F(1,23) = 5.89$, $p = 0.023$; the main effect of contrast type and the interaction effect were not significant (p's > 0.21). For the other three ROIs, no statistically-significant effect was observed (p's > 0.07).
